# Statistical tools for transgene copy number estimation based on real-time PCR

**DOI:** 10.1186/1471-2105-8-S7-S6

**Published:** 2007-11-01

**Authors:** Joshua S Yuan, Jason Burris, Nathan R Stewart, Ayalew Mentewab, C Neal Stewart

**Affiliations:** 1 Department of Plant Sciences, University of Tennessee, Knoxville, TN 37996, USA; 2 University of Tennessee Institute of Agriculture Genomics Hub, University of Tennessee, Knoxville, TN 37996, USA; 3 Department of Biology, Emory University, Atlanta, GA 30322, USA

## Abstract

**Background:**

As compared with traditional transgene copy number detection technologies such as Southern blot analysis, real-time PCR provides a fast, inexpensive and high-throughput alternative. However, the real-time PCR based transgene copy number estimation tends to be ambiguous and subjective stemming from the lack of proper statistical analysis and data quality control to render a reliable estimation of copy number with a prediction value. Despite the recent progresses in statistical analysis of real-time PCR, few publications have integrated these advancements in real-time PCR based transgene copy number determination.

**Results:**

Three experimental designs and four data quality control integrated statistical models are presented. For the first method, external calibration curves are established for the transgene based on serially-diluted templates. The Ct number from a control transgenic event and putative transgenic event are compared to derive the transgene copy number or zygosity estimation. Simple linear regression and two group T-test procedures were combined to model the data from this design. For the second experimental design, standard curves were generated for both an internal reference gene and the transgene, and the copy number of transgene was compared with that of internal reference gene. Multiple regression models and ANOVA models can be employed to analyze the data and perform quality control for this approach. In the third experimental design, transgene copy number is compared with reference gene without a standard curve, but rather, is based directly on fluorescence data. Two different multiple regression models were proposed to analyze the data based on two different approaches of amplification efficiency integration. Our results highlight the importance of proper statistical treatment and quality control integration in real-time PCR-based transgene copy number determination.

**Conclusion:**

These statistical methods allow the real-time PCR-based transgene copy number estimation to be more reliable and precise with a proper statistical estimation. Proper confidence intervals are necessary for unambiguous prediction of trangene copy number. The four different statistical methods are compared for their advantages and disadvantages. Moreover, the statistical methods can also be applied for other real-time PCR-based quantification assays including transfection efficiency analysis and pathogen quantification.

## Introduction

Transgenic organisms are produced by the introduction of exogenous DNA into the genome using techniques such as virus infection, transformation, injection, and particle bombardment [1]. Transgenic methods have emerged as a critical approach in biological and biomedical sciences with broad applications in gene function studies, biotechnology, and high throughput screening [1-3]. Transgene copy number is a key issue for transgenic studies since it is directly relevant to the effectiveness of transgenic event and data interpretation. Transgene copy number is defined as the number of exogenous DNA insert(s) in the genome. For example, if the exogenous DNA fragment inserts only once at a single locus of the genome, it is a single copy transgenic event. The copy number is closely relevant to another concept, zygosity. If a single exogenous DNA insert exists as two identical alleles on the homologous chromosomes in the organism, it is a homozygous transgenic line with one copy of transgene. If the single exogenous DNA insert exists in only one of the homologous chromosomes, it is a hemizygous (syn. heterozygous) transgenic line with one copy of transgene. Both transgene copy number and zygosity are important in the genetic analysis of gene function. Multiple transgene copies could lead to extremely high expression of the gene, and sometimes result in transgene silencing [4]. For insertional mutation studies where transposons are usually inserted into the endogenous gene to abolish the gene function, heterozygous transgene allele often leaves one functional copy of the gene, and homozygous transgene alleles is preferred for functional studies. For these reasons, transgene copy number and zygosity determination are usually an essential part of trasngene studies.

The conventional method for trangene copy number determination is Southern blot hybridization using two possible strategies. In the first strategy, restriction enzyme with only one restriction site in the transgene cassette will be chosen to digest the genomic DNA, and the digested DNA will be used for Southern blot hybridization with probes specific to transgene. If southern blot hybridization renders only one band, the transgene copy number should be one [5,6]. In the second approach, equal amount of genomic DNA from different transgenic lines will be digested with two restriction enzymes cutting regions flanking the transgene to render a fragment of an expected size, which is then hybridized with a transgene-specific probe. The resultant Southern hybridization signals are then normalized against those from serial dilutions of transgene and then compared to transgenic lines with known copy number to derive the copy number estimation [7]. Southern blot hybridization based transgene copy number determination is both costly, time consuming, and requires tens of microgram quantities of high-quality DNA. A robust alternative to Southern blot analysis would be helpful for gene function analysis and large scale mutant library based on transgenic techniques requiring a high-throughput determination of transgene copy number.

Real-time PCR has emerged the method of choice for fast, affordable, and efficient estimation of copy number [6,8-10]. Various platforms and efforts have been proposed to improve the accuracy of real-time PCR for this application using both the external standard curve based method and the ΔCt method involving an internal reference gene [8,11,12]. The details of several methods will be discussed in the result part, where statistical models were developed for these methods.

Despite the obvious advantages of real-time PCR, the accuracy and detection limit of real-time PCR based transgene determination has been controversial [10]. Mason et al. [10] found that only about 70% of the real-time PCR-based transgene copy determination results could be verified by Southern blot analysis. The copy number detection limit was also proposed to be 2 by previous research [5,6]. One limitation of previous research is the lack of thorough statistical analysis and proper models that specify copy number estimation for hypothesis testing. Explicit hypothesis testing has been seldom invoked for transgene copy number, and a predetermined and clear *P *value and confidence levels of estimation have not been specified; i.e. copy number = 1; *P *< 0.05. Among all the parameters, confidence levels are especially important for real-time PCR based transgene copy number determination, since it defines the precision and sensitivity of the assay. In case that confidence interval spans two integers, say, 1 and 2, it will be difficult to determine the actual copy number. Because of the limitations on statistical procedures, the results of analysis are often ambiguous and without clear confidence intervals. Moreover, robust quality control associated with real-time PCR analysis is also seldomly presented and has even been discouraged [13].

Recent advances in real-time PCR statistical analysis have provided an opportunity for improving real-time PCR-based transgene copy number estimation with rigorous quality control and robust hypothesis testing. In fact, one of the most prominent factors that are ignored in real-time PCR-based transgene copy number determination is amplification efficiency, for which a significant deviation from 100% can drastically alter copy number estimation. Amplification efficiency is therefore one aspect of quality control that must be addressed by statistical models [14,15]. Overall, the statistical models with a specified test for transgene copy number and integrated quality control for amplification efficiency will provide a badly needed solution toward well-defined transgene copy number estimation with confidence intervals and a probability value.

In this paper, we present several statistical models for the estimation and hypothesis testing of transgene copy number based on external standard curve or internal reference gene methods. For external standard curve methods, the Ct numbers for transgenic and control plant samples are fit into a simple linear regression model to estimate the copy number difference between the transgenic sample and a sample of known transgene copy number. For the internal reference gene-based method with a standard curve, we have developed a multiple regression/ANOVA model to estimate transgene copy number or zygosity based on the copy number of an endogenous reference gene, which is normally 2. We also present two additional models for real-time PCR-based transgene copy number determination using fluorescence data. For all of the models, the data quality control issues are discussed and integrated into the statistical models. Overall, the paper presents a set of quality control integrated statistical methods for transgene copy estimation, which allows a more precise and accurate estimation of transgene copy number with real-time PCR.

## Results

**Figure 1:**
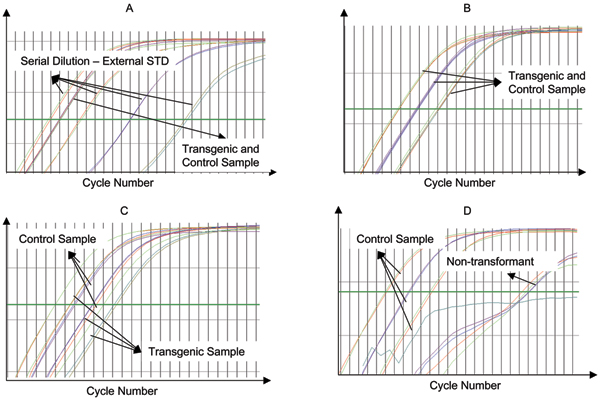
Representative real-time PCR results for transgene copy number quantification. A, The amplification plot for external standard curve method, where both serial diluted external control samples and transgene sample amplification curves are shown. B. The amplification plot for internal reference gene method, where transgene copy number equals to that of reference gene as shown by the overlapped amplification curves. C, The amplification plot for internal reference gene method, where transgene copy number does not equal to that of reference gene as shown by the separated amplification curves. D. The amplification plot for internal reference gene method, where non-transformants were tested as shown by the extremely large Ct numbers for transgene.

Both homozygous and hemizygous progenies for two transgenic lines were analyzed for transgene copy numbers. The schema of these analyses can be found in Figure 1. Statistical methods are developed for transgene copy number determination with external standard curve experimental design and internal standard curve experimental design, respectively. For internal standard curve data, models were developed for analyzing both Ct value and fluorescence data.

### Statistical methods for external standard curve experimental design

**Figure 2:**
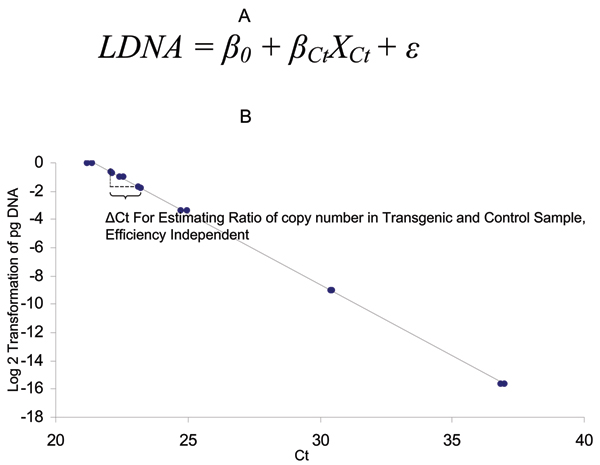
Statistical model for transgene copy number determination with external standard curve method. A. The simple linear regression model with LDNA (logarithm transformed input DNA amount) as dependent variable, and Ct number as independent variable. The external standard curve can be used for calculating input DNA amount from transgenic and control samples. The regression slope can be used for data quality control to estimate amplification efficiency. B. The linear regression as shown by plot of logarithm 2 based transformation of initial DNA input against Ct number.

The external standard curve design estimates the transgene copy number by comparison between a test sample and a control sample based on an external standard curve (Figure 2B). The standard curve based on simple linear regression can be first generated with logarithm-2 transformed initial DNA input as dependent variable and the Ct number as independent variable (Figure 2A). The simple linear regression can be used to estimate the amplification efficiency for quality control purpose as reported by Yuan et al [14,15]. Then, Ct numbers from control samples and transgenic testing samples were used to fit into the linear regression to derive the estimation of logarithm-2 transformed DNA amount for the transgene. Based on the estimated logarithm transformed DNA amount, a two group T-test can be carried out to generate the point estimation and confidence intervals for the ratio of DNA amount between the control sample and testing sample. Considering that the input genomic DNA is the same, the two group mean difference indicates the logarithm-2 transformed ratio of transgene copy number between control and testing transgenic samples.

A SAS program (Additional File 1) has been developed for analyzing a sample dataset (Additional File 2). The highlight of the program is as follows. The Procedure Regression in SAS was first executed to model Ct number against logarithm transformed input DNA concentration. The test statement for Ct's equivalence to -1 helps to estimate if the slope of the simple linear regression is -1 for data quality control purpose. As described by Yuan et al. 2007 [14], since logarithm-2 transformed initial DNA input is in reverse proportion to the Ct number, the slope of the simple linear regression is the negative amplification efficiency. In other words, if the slope equals to -1, the amplification efficiency is 100%. According to the test results, the amplification efficiency is not significantly different from 100% as shown by the *P *value of 0.62, and we can then proceed to analyze ratio of transgene copy number between the test sample and the control sample. In fact, since logarithm transformed DNA amount will be derived from the standard curve, which has already taken into the consideration of efficiency, the amplification efficiency testing is recommended but not necessary for the external standard curve design.

The output dataset from the regression procedure will contain the predicted logarithm transformed DNA amount for both control and test sample. The dataset is then cleaned up to include only control and test sample data. The predicted logarithm transformed DNA amount are then compared using a two-group student T-test with SAS Procedure Ttest. The output is as shown in Table 1 (Line 1 Heterozygous Method 1), and it should be noted that the mean difference estimation is control sample subtracting testing sample. The estimated values should be reversed, where the confidence interval covers -1, which is 0.5-fold as indicated by 2^-1^. Therefore, we determine that the transgenic sample transgene copy number is half of the control sample. In this case the control sample is from a homozygous plant with a single copy insert, and we are testing the transgenic sample for zygosity. Thus, we determine that the test sample is from a heterozygous plant with one transgene copy.

**Table 1:** Transgene copy number determination by different methods.

Line	Zygosity	Method	LgRt(T/C)	Copy Num	Confidence Interval	Efficiency Adjust	Pred Copy	Exp Copy
1	Hemi-zygous	1	-1.05	0.49	(0.42, 0.56)	N/A	0.5	0.5
		2	-1.07	0.48	(0.33, 0.68)	No	0.5	0.5
		3	-0.93	0.53	(0.32, 0.42)	Yes (0.93)	0.5	0.5
		4	-1.00	0.5	(0.12, 2.23)	N/A	N/A	0.5
	Homo-zygous	1	-0.10	0.93	(0.79, 1.09)	N/A	1	1
		2	-0.06	0.96	(0.77, 1.19)	No	1	1
		3	0.01	1.01	(0.85, 1.21)	No	1	1
		4	-0.80	0.57	(0.24, 1.38)	N/A	N/A	1
2	Hemi-zygous	1	-1.10	0.47	(0.41, 0.52)	N/A	0.5	0.5
		2	-1.18	0.44	(0.21, 0.95)	No	0.5	0.5
		3	-1.25	0.42	(0.33, 0.52)	No	0.5	0.5
		4	0.06	1.04	(0.22, 4.99)	N/A	N/A	1
	Homo-zygous	1	0.19	1.14	(0.93, 1.40)	N/A	1	1
		2	-0.03	0.98	(0.81,1.19)	No	1	1
		3	0.18	1.13	(0.88, 1.47)	Yes (0.97)	1	1
		4	0.09	1.06	(0.31, 3.68)	N/A	N/A	1

The transgene copy numbers for both homozygous and hemizygous progenies of two transgenic lines were analyzed with real-time PCR and different statistical methods. Method 1 is the external standard curve design, Method 2 is the internal reference gene design with standard curve, Methods 3 and 4 are the internal reference gene design with fluorescence data. In Method 3, the cycle number was used as dependent variable, and in Method 4, the logarithm-transformed fluorescence signal was used as the dependent variable. Column 4 shows the logarithm-2 transformed ratio between the test line and control line, or between the transgene and internal reference gene. Column 4 presents the direct outputs from SAS code. Column 5 indicates the numeric estimation of copy number. The confidence levels for logarithm 2 transformed ratios are converted into confidence interval for transgene copy number and presented in Column 6. Column 7 indicates whether efficiency adjustment was included or not. The predicted and actual copy numbers were presented in Column 8 and 9.

Overall, simple linear regression combined with a two-group T-test can be used to model real-time PCR data for transgene copy number determination based on external control curve. The similar strategy can also be used for broad applications of absolute quantification, transfection efficiency assay and other applications.

### Statistical methods for internal reference gene design with standard curve

The external standard curve-based transgene copy number estimation is one of the earliest proposed strategies. However, the strategy has two major limitations. First of all, it requires an external control sample with known transgene copy number; second, the strategy heavily depends on the accuracy of DNA measurement, since both samples will need to have exact amount of input DNA. These often-practical limitations have inspired researchers to adopt the internal reference gene-based method for transgene copy number determination. The approach involves an internal reference gene with known copy number, and compares the transgene copy number with the internal reference gene to estimate the copy number of transgene [6,16]. The advantage of this approach is that it does not rely on the accuracy of DNA measurements and does not require a control plant sample with known copy number. However, the successful application of the method is contingent on data quality control; e.g., amplification efficiencies. This method has been used with various success and failure. Some researchers found an almost 90% of correlation between Southern blot and real-time PCR transgene copy number estimation, whilst others observed less than 50% accuracy [6,8,16]. This inconsistency highlights the more rigorous data quality control requirements for the method, especially in terms of amplification efficiency, since no DNA amount was calculated in this design and the quantification is based on ΔCt. The amplification efficiency consideration is acutely important for between-gene standardization. For the internal reference gene design, the amplification efficiency for both the reference gene and transgene should be equal. Moreover, the amplification efficiency for both genes should be not significantly different than 100%. If these prerequisites cannot be met, more complicated amplification efficiency adjusted models should be applied as described by Yuan et al. [14,15]. Standard curve design is therefore recommended to be included in the experimental design for amplification efficiency calculation and the data quality control.

**Figure 3:**
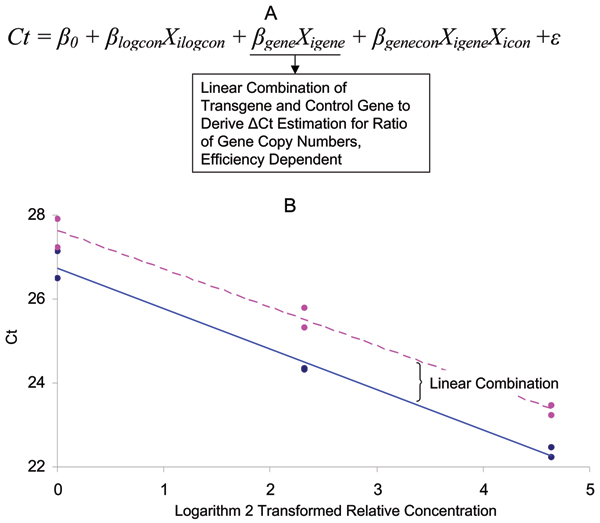
Statistical model for transgene copy number determination with internal reference gene design based on standard curve. A. Two-way ANOVA model with Ct number as dependent variable, and logarithm transformed concentration (logcon) and gene category (transgene or reference gene) as independent variables. The model can be used to derive ratio of copy number between transgene and reference gene and to test the equal amplification efficiency. B. The regression view of Ct plotted against logarithm 2 based transformation of relative concentration of genomic DNA template for both transgene and reference gene. Pink line is for transgene, and blue line is for control gene.

A two-way ANOVA model or multiple regression model (Figure 3A) will be able to derive the estimation of transgene copy number based on the internal reference gene. The model can also be considered as an ANCOVA model if the gene category variable is considered as covariate. However, ANCOVA model usually strictly requires equal amplification efficiency (equal slope for regression) between the samples, whereas ANOVA and multiple regression model are more flexible. A regression view of the model can be seen from Figure 3B, where the slope for each gene can be used estimate the amplification efficiency. When amplification efficiencies are not significantly different from 100% for both genes, we can use linear combination of transgene Ct number and reference gene Ct number to derive the ratio of copy number between the transgene and reference gene. The null hypothesis is that transgene and reference gene have the same copy number, and the alternative hypothesis is that the transgene and reference gene copy numbers are different. The linear combination estimation of mean differences will render the ΔCt, indicating the logarithm 2 transformed copy number difference between transgene and reference gene. Since most of the reference genes are homozygous single copy, a -1 in linear combination will indicate the heterozygosity of transgenic sample. If the linear combination renders a 0, it indicates a homozyous transgenic sample with one copy of transgene. A linear combination results higher than 1 will indicate multiple copies.

A test dataset for the internal reference gene design and a SAS program for analyzing the dataset are included in Additional Files 3 and 4, respectively. In this case, we are testing the zygosity of a transgene in tobacco. A similar data quality control step is included to test if each gene's amplification efficiency is 100%. Then the logarithm-2-based ratio of transgene and reference gene copy number is derived from linear combination (Figure 3A). The results are summarized in Table 2 (Line 1 Heterozygous Method 2). The output for linear combination of the ΔCt equals to -1.07, which translates to 0.5 times of reference gene. It clearly indicates that the transgenic sample is heterozygous. Another important aspect of the result is the confidence interval, which is between -1.60 and -0.56 in this study. It translates into confidence intervals of transgene to reference gene ratio = 0.33 to 0.68. Since the transgene copy number should be multiple scales of 0.5 (0.5, 1, 1.5, etc.), the confidence interval covering 0.5 indicates that it is not significantly different from 0.5 with our *P *value cutoff of 0.05. Overall, the result indicates that the transgenic sample has one copy of transgene and it from a hemizygous plant. In addition to the above data, several other transgenic lines are analyzed and the results are presented and compared in Table 2.

**Table 2:** The primer sequences for the real-time PCR experiments. The gene specific primer for tobacco (*Nicotiana tabacum*) *NtWBC1 *gene and *Arabidopsis AtWBC19 *gene were designed as follows.

Gene Name	Forward Primer	Reverse Primer
*NtWBC1*	ATCTCACGTAGCCGGAGCA	TTTGTTCTGGTGGACGGGAT
*AtWBC19*	AAGAAACGCGGCGAACAC	GCCGTCTCTCTCCTCCAGAA
*AtWBC19*	TTAGCCAAACGGTACATGAAAAAC	CCAGTCACCATTACCGTAGCAA

### Statistical methods for internal reference gene design based on fluorescence data

Besides the standard curve data, fluorescence output from real-time PCR experiment can also be modeled to derive the ratio of copy number between transgene and reference gene. Even though the accuracy of using fluorescence output to estimate the transgene copy number is still a controversial topic, some researchers would argue that analyzing amplification efficiency and gene quantification with fluorescence data can avoid the complexity of standard curve design [14].

**Figure 4:**
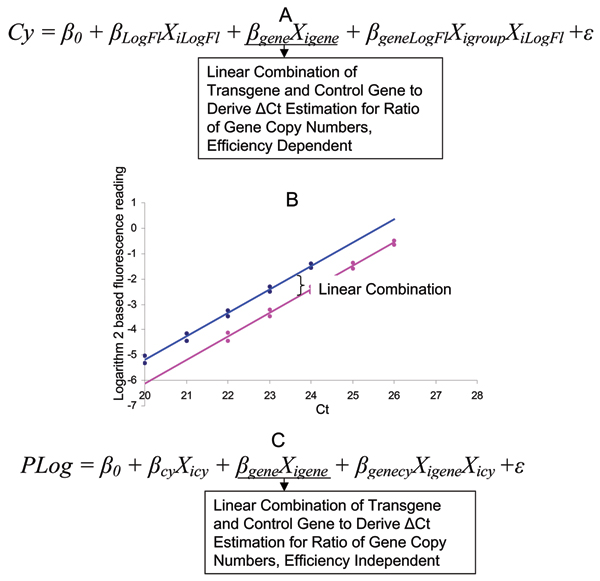
Statistical models for transgene copy number determination with internal reference gene design and fluorescence data. A. Two-way ANOVA model with cycle number (Cy) as dependent variable, logarithm transformed fluorescence signal (LogFl) and gene category (transgene or reference gene) as independent variables. The model can be used to derive the ratio of copy number between transgene and reference gene and to test the equal amplification efficiency. B. The regression plot of logarithm-2-based fluorescence signal strength against cycle number for both transgene and control gene. The pink line corresponds to the transgene, and blue to the control gene. C. The multiple regression model with logarithm transformed fluorescence (LogFl) as dependent variable, and cycle number (Cy) and gene category as independent variables. The model is efficiency integrated and can be used to derive the ratio of copy number between transgene and reference gene based on linear combination of intercept.

There are two ways to model the fluorescence data for ΔΔCt method of gene expression analysis as shown by Yuan et al. [14]. Similarly, we can also model transgene copy number data using two different approaches as shown in Figure 4A and 4C. For the first model (Figure 4A), it is similar to the model used in previous part for the ΔCt data analysis. It has the same amplification efficiency prerequisites as the previous model. The amplification efficiency estimation and equivalence test can be performed as described by Yuan et al., 2007 [14]. The implementation of the model using SAS code is similar to that for the ΔCt data, and the test dataset and SAS program are included in Additional Files 5 and 6. The data in Additional File 5 can be derived from selecting the fluorescence signal from exponential phase of PCR with different methods [14]. The SAS program is similar to that described above, except that cycle number was used for modeling the logarithm 2-based transformed fluorescence data. No Ct number is necessary for the model. Moreover, we used 0.93, the pooled amplification efficiency, for the efficiency adjustment as described by Yuan et al. [14,15]. As shown in Table 2 (Line 1, Hemizygous, Method 3), the output of the analysis clearly indicates that the sample is hemizygous as indicated previously.

An alternative model (Figure 3B) has been used for gene expression analysis in previous studies [14,17]. The model uses the linear combination of the intercept from a multiple regression model with logarithm transformed fluorescence data as the dependent variable, and cycle number and gene as the independent variables. The analysis assumes that intercept represents the original template amount and the linear combination can derive the ratio of copy number between the reference gene and transgene. The model will use the same fluorescence data as the previous model without the efficiency adjustment since amplification efficiency is already integrated into the model. Additional File 6 contains the SAS program that can analyze Additional File 5 for the model as shown in Figure 4B and 4C. The output of the analysis is shown in Table 2 for Line 1 Method 4 with a hemizygous plant, which has a similar point estimate, yet a larger error as compared to the other methods described here.

Overall, linear models can be used for modeling real-time PCR data for transgene copy number determination, which will lead to accurate estimation of copy numbers as well as precise estimation of errors and confidence levels. Robust data quality control can be integrated in the models to improve the accuracy of the analysis.

### Verification of transgene copy number by Southern blot and segregation experiments

Single copy transgene numbers were determined using Southern blot analysis for both transgenic lines as previously described [7], line 1 and 2 corresponds to line 28 and 30 in our previous publication [7]. The zygosity was confirmed by genetic analysis of progeny based on kanamycin resistance, with a 3:1 ratio of segregation indicating the parent of this selfing plant was hemizygous. In fact, the segregation experiments also confirmed the transgene copy number was equal to one.

## Discussion

### Importance of statistical models for real-time PCR-based transgene studies

Previous studies estimated transgene copy number with real-time PCR based on the point estimation of ratio between transgene and reference gene. However, appropriate statistical models are necessary to provide point estimation and confidence intervals for analyzing both transgene copy number as well as the error associated with the analysis. Moreover, robust data quality control needs be integrated in the statistical models to improve the accuracy of the estimation.

The interpretation of confidence interval is important. Considering zygosity, the transgene copy number should be multiple times of 0.5 as compared to reference gene. In other words, a hemizygous, one-copy transgene will be 0.5 times of the reference gene. A homozygous one copy transgene will be one (2 by 0.5) times the reference gene. A homozygous two copy transgene will be two (4 by 0.5) times of reference gene. The confidence intervals, therefore, can be interpreted as the confidence that a certain *P *value (normally 0.05) accurately determines whether a transgene number is significantly different from a predetermined test number, which should be multiple times of 0.5. For example, if the 95% confidence interval of a test result is between 0.25 and 0.75, then the testing result is that the transgene copy number is not significantly different from 0.5 at a *P *value of 0.05, which indicates the sample came from a hemizygous single copy transgenic plant.

From the results, we can observe congruent results between real-time PCR and the Southern blot analysis, which indicates the potential of real-time PCR to be an efficient and precise alternative for transgene copy number determination. Moreover, previous studies indicate that the real-time PCR based transgene copy number determination has a detection limit of 2 copies [5]. Statistical models provide the potential of increasing not only the detection limits but also precision of detection.

### Comparison of different statistical models

From Table 2, we can see that all of the four statistical methods render similar point estimation for all four transgenic lines analyzed. However, the last model analyzing intercepts of fluorescence data renders a larger confidence interval and standard error, which leads to an ambiguous estimation of copy number. For all the three other methods, the copy number estimation is precise. The error overestimation for intercept-based analysis has been discussed previously by Yuan et al. [14]. It seems that the other three methods are better choices than the intercept-based analysis. Moreover, as discussed previously, the internal reference gene-based method generally is better than the external standard curve method since it does not require equal amounts of input DNA between the control and transgenic samples. However, the internal reference gene methods require more robust data quality control, especially for amplification efficiency. The external standard curve method is efficiency independent since it calculates the DNA amount for ratio estimation.

### Data quality control

Data quality control includes an amplification efficiency test and dissociation curve. It should be noted that equal amplification efficiency between the reference gene and transgene is a prerequisite for the internal reference gene design. Even though efficiency adjustment can be conducted for gene expression analysis, the practice of unbalanced linear combination-based adjustment for transgene copy number determination might introduce another source of experimental error [14]. The precise transgene copy number determination assumes the confidence interval to be smaller than 1 for an accurate estimation of transgene copy number. For example, a larger confidence interval of (0.4, 2.1) will lead to no conclusive estimation of copy number. It is therefore important to minimize the error introduced by either unequal amplification efficiency or low amplification efficiency. In the best scenario, all PCR reactions should have amplification efficiency equal to 100%. However, it is also acceptable that all of the reactions have equal amplification efficiency, but less than 100%. During the linear combination analysis of ratio of copy number, the amplification efficiency can be integrated into the statistical model. The standard curve method is therefore preferred since it can help to determine the amplification efficiency more precisely [14].

### Other applications of statistical models

The proposed statistical models can be used for a variety of other real-time PCR based quantification analysis. The external standard curve method can be used for absolute quantification of real-time PCR data, which has been widely used for gene expression or gene copy number analysis. The other models can also be applied for transfection assay efficiency estimation, large transgenic library screening, or pathogen quantification.

## Methods

### Plant growth, plant transformation, segregation experiments, DNA extraction, and Southern blot analysis

Transgenic tobacco plants are described elsewhere [7]. Briefly, leaf disk transformation experiments were performed using *Agrobacterium tumefaciens *strain GV3850 and *Nicotiana tabacum *cv. Xanthi [1]. Transformants were selected using 50, 100, or 200 mg/l kanamycin monosulfate (Sigma-Aldrich, St. Louis, MO) and 200 mg/l timentin (GlaxoSmithKline, Bucks, UK) in triplicate. Transgenic tobacco plants (T_0_) were then grown to obtain T_1 _seed. In order to generate homozygous plants, segregation experiments were carried out by growing T_1 _seeds on Murashige and Skoog (MSO) media containing 200 mg/l kanamycin [18]. Seeds were surface sterilized and placed onto MSO plates, and were observed for a 3:1 segregation, indicating hemizogosity. To obtain T_2 _seed, six plants of each transgenic type were pulled from each plate and grown in the greenhouse. This generation was then placed on MSO containing 200 mg/l kanamycin to confirm homozygous plants.

Both Southern blot analysis and real-time PCR were carried out to confirm the gene copy number. Southern blot analysis was performed as described by Mentewab et al. [7]. Briefly, genomic DNA was extracted from the leaf tissue of T_0 _tobacco plants and 10 μg was digested with *Kpn*I and *Sac*I to obtain a 2.2 kb fragment found in transgenic events containing the coding region of the *Atwbc19 *gene. Digested DNA was separated using a 0.7% (wt/vol) agarose gel and blotted onto Hybond-Ny (Amersham Biosciences, Buckinghamshire, UK). The *Atwbc19 *fragment was obtained by digesting pNPT-ABC with *Kpn*I and *Sac*I, radiolabeled with [^32^P] using random primers and RadPrime DNA labeling technology according to manufacturer's instructions (Invitrogen, Carlsbad, CA). A Personal FX phosphoimager (BioRad, Hercules, CA) was used to capture the autoradiographic image.

### Real-time PCR experiments and primer design

Genomic DNA was diluted into 20 ng/ul and three 1:5 serial dilutions were made. Duplicated real-time PCR experiments were performed for each dilution with an ABI 7000 Sequence Detection System (Applied Biosystems, Foster City, CA) and SUPER SYBR Mix (Applied Biosystems, Foster City, CA). The PCR primers were designed by PrimerExpress (Applied Biosystems, Foster City, CA), and the primer pairs are shown in Table 1. The real-time PCR experiments were conducted using a slightly modified manufacturer's protocol as outlined below. A two minute 50°C initial step was followed by a ten minute 95°C step to activate the Taq polymerase. The next 45 cycles were carried out with 25 seconds of 95°C followed by 80 seconds of 60°C. The experiments were analyzed with auto-baseline and manual threshold chosen from the exponential phase of the PCR amplification. After the data analysis, the Ct number and DeltaRn were exported for statistical analyses.

In order to generate the external standard curve for transgene copy determination, PCR products were generated with the same primer pairs from genomic DNA and purified with gel purification kit (Qiagen Inc., Valencia, CA). Standard curves were generated with 5 serial dilutions ranging from approximately 0.00002 pg to 1 pg of DNA template. For internal reference gene design, the real-time PCR reactions were carried out as described above for both transgene (*AtWBC*) and internal reference gene (*NtWBC*) with primers shown in Table 2. Two parallel pairs of primers were designed for *AtWBC*.

### Program development

All programs were developed with SAS 9.1 (SAS Institute, Cary, NC).

## Conclusion

Overall, we presented four statistical models for three experimental designs of real-time PCR-based transgene copy number determination. Recent progress in real-time PCR data analysis are integrated for better data quality control and confidence interval estimation. The advantages and disadvantages of each statistical model were discussed. Three out of four models render similarly good results, and the internal reference gene design is particularly recommended for its independence of external control sample and DNA quantification requirements. The SAS programs and guidelines for practical applications were included. The methods presented here provide a solution for more precise transgene copy number estimation based on real-time PCR. These methods can also be applied to broader aspects of real-time PCR based quantification.

## Competing interests

The authors declare that they have no competing interests.

## Authors' contributions

JSY carried out DNA extraction, real-time PCR experiments, statistical modeling, SAS programming. JSY also drafted most of the paper and produced the displays. JB contributed to plant transformation experiments and performed plant growth and segregation experiments. JB also drafted part of the method for the paper. NRS helped with multiple tasks in the UTIA Genomics Hub including this work. AM performed the Southern blot and performed supporting work with the transgenic plants. CNS Jr. proposed the work and finalized the draft.

## Supplementary Material

Additional file 1 SAS program. SAS program to analyze data in additional file number 2 Click here for file

Additional file 2 dataset. Data analyzed by SAS program in additional file number 1 Click here for file

Additional file 3 dataset. Data analyzed by SAS program in additional file number 4. Click here for file

Additional file 4 SAS program. SAS program to analyze data in additional file number 3. Click here for file

Additional file 5 dataset. Data analyzed by SAS program in additional file number 6. Click here for file

Additional file 6 SAS program. SAS program to analyze data in additional file number 5. Click here for file
